# MDF2Former: Multi-Scale Dual-Domain Feature Fusion Transformer for Hyperspectral Image Classification of Bacteria in Murine Wounds

**DOI:** 10.3390/jimaging12020090

**Published:** 2026-02-19

**Authors:** Decheng Wu, Wendan Liu, Rui Li, Xudong Fu, Lin Tao, Yinli Tian, Anqiang Zhang, Zhen Wang, Hao Tang

**Affiliations:** 1 School of Automation, Chongqing University of Posts and Telecommunications, Chongqing 400065, China; wudc@cqupt.edu.cn (D.W.); liuwendan@yeah.net (W.L.); lirui@cqupt.edu.cn (R.L.); al2o24ever@gmail.com (X.F.); lintao_cqupt@yeah.net (L.T.); 2 School of Computer Science, Chongqing University of Posts and Telecommunications, Chongqing 400065, China; yltian@cqupt.edu.cn; 3 State Key Laboratory of Trauma and Chemical Poisoning, Intensive Care Unit, Daping Hospital, Army Medical University, Chongqing 400042, China; zhanganqiang@tmmu.edu.cn (A.Z.); dpicuwz@tmmu.edu.cn (Z.W.)

**Keywords:** wound bacteria identification, hyperspectral imaging (HSI), convolutional neural networks (CNN), Transformer, deep learning

## Abstract

Bacterial wound infection poses a major challenge in trauma care and can lead to severe complications such as sepsis and organ failure. Therefore, rapid and accurate identification of the pathogen, along with targeted intervention, is of vital importance for improving treatment outcomes and reducing risks. However, current detection methods are still constrained by procedural complexity and long processing times. In this study, a hyperspectral imaging (HSI) acquisition system for bacterial analysis and a multi-scale dual-domain feature fusion transformer (MDF2Former) were developed for classifying wound bacteria. MDF2Former integrates three modules: a multi-scale feature enhancement and fusion module that generates tokens with multi-scale discriminative representations, a spatial–spectral dual-branch attention module that strengthens joint feature modeling, and a frequency and spatial–spectral domain encoding module that captures global and local interactions among tokens through a hierarchical stacking structure, thereby enabling more efficient feature learning. Extensive experiments on our self-constructed HSI dataset of typical wound bacteria demonstrate that MDF2Former achieved outstanding performance across five metrics: Accuracy (91.94%), Precision (92.26%), Recall (91.94%), F1-score (92.01%), and Kappa coefficient (90.73%), surpassing all comparative models. These results have verified the effectiveness of combining HSI with deep learning for bacterial identification, and have highlighted its potential in assisting in the identification of bacterial species and making personalized treatment decisions for wound infections.

## 1. Introduction

Wound infection constitutes a critical challenge in trauma repair and clinical therapy. It not only significantly delays the healing process of the wound, but in severe cases, it may even lead to fatal complications such as sepsis and multiple organ failure [1,2]. According to World Health Organization estimates, burn injuries affect 11 million people annually worldwide, causing approximately 180,000 deaths and substantial morbidity, with bacterial infection being one of the principal etiologies [3]. Consequently, achieving rapid and accurate bacterial detection carries significant clinical implications for guiding appropriate antibiotic therapy and mitigating the risk of severe outcomes.

The traditional bacterial detection methods can be broadly classified into three categories based on their detection mechanisms and information sources: methods based on morphological observation, analytical methods based on physicochemical or biological reaction characteristics, and detection methods based on molecular biological signals. Among these, morphological-related methods mainly rely on the morphological and growth characteristics of bacteria under microscopic observation or in culture conditions, such as smear microscopy and colony morphology analysis [4]. This type of method usually requires manual interpretation, and the results are highly dependent on the operator’s experience, with limited ability to distinguish in complex infection environments [5]. Methods related to physicochemical or biological reaction characteristics include chromatography [6], chemical colorimetry [7], and inductive impedance technology [8], which analyze by detecting bacterial metabolites or physical response signals, but often involve complex experimental procedures or specific detection conditions, and have a long detection cycle. Molecular biology and biosensor methods (such as PCR technology [9], gene chips [10], and biosensors [11]) achieve high-sensitivity detection by identifying specific nucleic acid sequences or biomolecules, but they rely on laboratory conditions and the detection results are greatly influenced by target selection and sample processing procedures [12]. Although traditional methods have played an important role in bacterial detection, they still have shortcomings in terms of detection speed, operational complexity, invasiveness, and the ability to characterize information in the wound area. Currently, there is an urgent need to develop a rapid, non-contact, and unlabeled bacterial identification method as an effective supplement to the traditional detection process.

In recent years, the development of optical detection technologies has provided new approaches for diagnosing wound infections. Hyperspectral image (HSI) captures hundreds of contiguous spectral bands, thereby furnishing rich spectral and spatial information [13]. Based on the unique advantages of HSI, such as high spectral resolution and the ability to conduct joint analysis of spectral and spatial information, scholars from both domestic and international communities have conducted a series of studies on its application in bacterial detection. Turra et al. [14] employed hyperspectral technology combined with soft independent modeling of class analogy (SIMCA) to achieve rapid differentiation of five types of urinary tract infection bacteria, including *Escherichia coli* and *Staphylococcus aureus*, on the culture medium. Bonah et al. [15] integrated visible-near-infrared (VIS-NIR) hyperspectral imagery with a support vector machine (SVM) classifier optimized by particle swarm optimization, achieving effective identification and differentiation of foodborne pathogens on culture media. Qiu et al. [16] developed an SVM classification model to detect *Staphylococcus aureus* in chicken breast using hyperspectral imaging qualitatively. Despite recent advances, current hyperspectral bacterial classification methods still struggle to exploit spectral-spatial information fully, necessitating more effective solutions.

Deep learning automatically and hierarchically extracts features by learning parameters, and has attracted increasing attention in computer vision tasks [17]. With advancements in deep learning, numerous deep-learning-based approaches have been applied to HSI classification to improve performance [18,19,20]. For instance, Kang et al. [21] proposed a long short-term memory (LSTM) network-assisted hyperspectral microscopic imaging (HMI) approach, which effectively distinguishes five common foodborne pathogens and achieves the highest accuracy compared to traditional machine learning methods. Liu et al. [22] proposed a simple convolutional neural networks (CNN) architecture that effectively classified dark septate endophyte (DSE) fungal isolates in hyperspectral images. Wu et al. [23] proposed a CNN-BiGTrans interactive aggregation network that can effectively classify commonly cultured wound bacteria.

Although the existing deep learning-based hyperspectral image classification methods have shown good performance in controlled bacterial recognition tasks, their applicability in the wound bacterial detection scenario is still limited. Wound infections have complex spatial morphology, subtle spectral differences among bacteria, and high-dimensional spectral redundancy, which pose significant challenges to current models. The existing CNN and Transformer methods still have shortcomings in the wound bacterial classification task: CNN is limited by a limited receptive field and is unable to represent spectral dependence; while Transformer sacrifices local spatial details and introduces high computational costs when dealing with high-dimensional data. Moreover, the existing methods tend to decouple spatial and spectral features, ignoring the joint interaction of spatial–spectral features in the wound context. Additionally, the existing hyperspectral bacterial classification research is mostly based on idealized experimental conditions such as culture media, lacking real wound data support, which limits the application verification and promotion of the methods. To address these limitations, this study has built a hyperspectral imaging acquisition system for collecting hyperspectral data of wound bacteria and proposed a multi-scale dual-domain feature fusion transformer framework (MDF2Former). Compared with traditional bacterial detection methods that rely on invasive sampling and time-consuming processing, the proposed method, as a rapid and non-invasive auxiliary identification module, enables in situ acquisition of spatial–spectral information from the entire wound surface and provides real-time bacterial species prediction, thereby offering a novel solution to support early clinical treatment decision-making. The principal contributions of this work are summarized as follows.

- A multi-scale dual-domain feature fusion transformer (MDF2Former) was proposed for the classification of hyperspectral images of bacterial infections on mouse wounds.
- In the model design, a multi-scale feature extraction module and a spatial–spectral dual-branch attention mechanism are introduced to enhance feature representation, while a dual-domain encoding mechanism jointly models spatial–spectral features in both the spatial and frequency domains.
- For the actual wound conditions, we established a hyperspectral imaging system to construct a mouse wound bacterial dataset containing eight bacterial species at four different concentrations (a total of 52,130 samples). The experimental results demonstrated that the proposed method performed exceptionally well in terms of classification performance and computational efficiency.

## 2. Related Work

### 2.1. CNN-Based Methods for HSI Classification

Research on convolutional neural networks (CNNs) for HSI classification has become quite extensive. Hu et al. [24] proposed a one-dimensional CNN (1D-CNN) that efficiently extracts local spectral features through multiple convolutional layers. Subsequently, Zhao and Du [25] introduced a two-dimensional CNN (2D-CNN) for extracting spatial–spectral features and adopted balanced local discriminant embedding. Later, Ben Hamida et al. [26] developed a three-dimensional CNN (3D-CNN) to extract joint spatial–spectral features from HSI cubes directly. The 1D-CNN excels at directly processing spectral sequences, while the 2D-CNN focuses on spatial feature extraction and is adept at capturing local spatial structures in images. The 3D-CNN directly models the cross-domain associations between spatial and spectral dimensions. Since each CNN type offers unique advantages in HSI feature representation, recent studies often employ hybrid networks combining them for HSI classification. Hao et al. [27] proposed an in vivo human brain HSI classification method that combines multiple deep models. This method first extracts spectral features using a 1D deep neural network (1D-DNN), and then employs a 2D-CNN for spatial–spectral feature extraction. Roy et al. [28] introduced the HybridSN network, which utilizes a 3D-CNN to extract spatial–spectral features, followed by a 2D-CNN to capture additional spatial features. Similarly, Ghaderizadeh et al. [29] applied a hybrid 3D-2D CNN for hyperspectral image classification, achieving better performance than using a single CNN. Cai et al. [30] proposed a attention-based triple-stream fused CNN (ATSFCNN), which overcomes the limitations of single-stream approaches by fusing features from 1D, 2D, and 3D CNNs, enabling more comprehensive information extraction. However, such CNN still rely on fixed receptive fields and local convolution operations, which limit their ability to capture long-range spectral dependencies and global contextual relationships in hyperspectral images.

To enhance the feature representation capability of CNNs in HSI classification, attention mechanisms have been integrated into CNN frameworks. These mechanisms help models learn more discriminative features by adaptively suppressing redundant information and enhancing salient features. Xue et al. [31] designed a hierarchical residual network with an attention mechanism (HResNetAM), which assigns adaptive weights to spatial and spectral features at different scales, further improving the discriminability of the extracted features. Yang et al. [32] proposed a cross-attention spectral-spatial network (CASSN), which utilizes both local and global spectral information of pixels to generate band-wise weights for suppressing redundant spectral bands. Moreover, most attention-enhanced CNNs primarily focus on either spatial or spectral weighting, without considering their interactions, which is suboptimal for complex wound environments where spectral responses and spatial morphology are strongly coupled.

Although the existing hyperspectral image classification methods based on convolutional neural networks have demonstrated excellent performance, when applied to the identification of wound bacteria, they still have inherent limitations, especially in terms of simulating long-range spectral correlation and global spatial–spectral interactions, where they perform poorly.

### 2.2. Transformer-Based Methods for HSI Classification

With the remarkable success of the vision transformer (ViT) [33] in the field of computer vision, Transformer networks have been widely adopted in HSI classification tasks due to their outstanding ability to model global, long-range dependencies. Liao et al. [34] proposed a spectral-spatial fusion transformer network (S2FTNet) for HSI classification. S2FTNet employs a transformer framework to construct spatial and spectral transformer modules, capturing long-range dependencies in both spatial and spectral domains. Hong et al. [35] approached HSI classification from a sequential perspective using transformer, and introduced a novel backbone network called SpectralFormer, which learns local spectral sequence information from adjacent bands to generate grouped spectral embeddings. Zhou et al. [36] designed the RNN-Transformer (RT) model by incorporating a positional embedding strategy that integrates ranking bias and attention redistribution, enhancing sequence-level features. Yang et al. [37] proposed a center-to-surround interaction learning (CSIL) framework, which utilizes a carefully designed hierarchical resampling strategy, a center transformer, and a surrounding transformer to enable multi-scale, detail-aware, and spatially interactive classification. Qiu et al. [38] developed a cross-channel dynamic spatiotemporal fusion transformer (CDSFT), which uses transposed multi-head self-attention to extract cross-channel global features and introduces a dynamic feature enhancement module and a positional attention module to enhance joint spectral-spatial feature learning for classification. However, such Transformer-based models prioritize global dependency modeling at the expense of local spatial details. Consequently, they exhibit insufficient granularity in feature extraction when characterizing diminutive bacterial colonies with irregular morphologies in wound images.

To address this limitation, Mei et al. [39] proposed the group-aware hierarchical transformer (GAHT), which introduces a novel grouped pixel embedding module to emphasize local relationships among spectral channels, thereby enabling the joint modeling of both global and local spatial–spectral relationships. Zou et al. [40] presented the local-enhanced spectral-spatial transformer (LESSFormer), which explicitly reinforces local information through a simple attention mask to enhance the representation of labeled samples. Xu et al. [41] proposed the spatial–spectral 1D swin transformer (SS1DSwin), which learns spatial–spectral relationships from both local and hierarchical perspectives and fuses them via cross-block normalization connections. Qiao et al. [42] introduced a novel multi-scale neighborhood attention transformer (MSNAT) that emphasizes neighborhood pixels within local window sizes and extracts multi-scale spatial information using various window sizes. However, as the computational complexity of the self-attention mechanism grows quadratically with the sequence length, it poses significant computational challenges when processing hyperspectral data containing hundreds of spectral bands.

To address these limitations, we propose a deeply fused CNN-Transformer cascade architecture that enables collaborative modeling of local and global relationships through a hierarchical feature learning mechanism. Specifically, CNN is used as the front-end for feature extraction, pre-extracting pixel-level multi-scale spatial and spectral features. These features are embedded into a dual-branch spatial–spectral attention module, where dynamic and adaptive weight assignment is employed to compress the feature dimensions, thereby reducing the input sequence length for the transformer and alleviating computational burden. Additionally, convolutional operations are integrated into the Transformer’s self-attention layers to form a hierarchical learning mechanism combining local convolution and global attention.

## 3. Proposed Network

### 3.1. Overall Network Architecture

**Figure 1:**
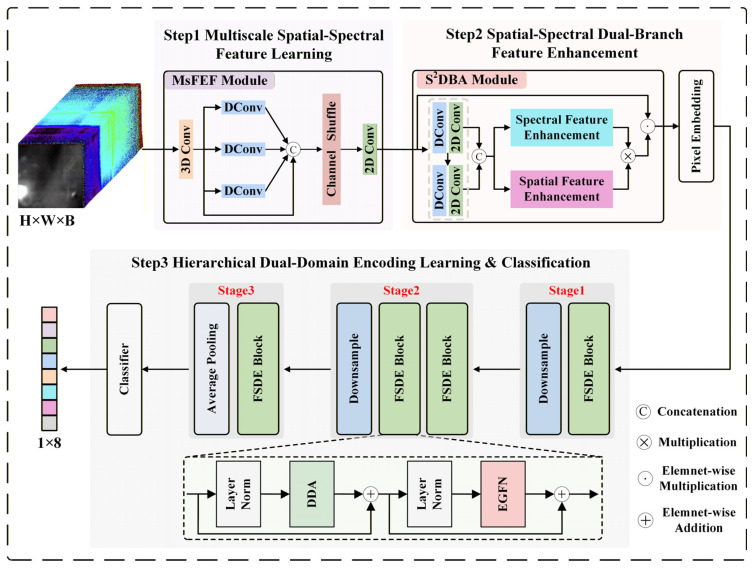
The proposed MDF2Former model framework.

The architecture of the proposed multi-scale dual-domain feature fusion transformer (MDF2Former) is illustrated in Figure 1. MDF2Former is designed as a CNN-Transformer-based network to extract and model multi-scale spatial–spectral information from HSIs effectively. The model decomposes the complex bacterial classification task into three interrelated feature learning steps: Step (1) employs the multi-scale feature enhancement and fusion (MsFEF) module to process the input HSI in order to capture multi-scale spatial–spectral features. Multi-scale convolutions are employed to extract discriminative representations at different receptive fields. Step (2) employs the spatial–spectral dual-branch attention (S^2^DBA) module to dynamically select and collaboratively enhance spatial regions and their corresponding spectral signatures, while pixel embedding is used to encode the features into tokens. Step (3) employs hierarchically stacked frequency and spatial–spectral domains encoding (FSDE) modules to model the interactions between global and local tokens. In the first two stages, feature maps are progressively down-sampled to generate multi-scale tokens, whereas in the final stage, global average pooling is applied to aggregate the spatial dimensions. Finally, a fully connected layer is used to produce the classification results. The detailed structures will be described in the following subsections.

### 3.2. Multi-Scale Spatial–Spectral Feature Learning

**Figure 2:**
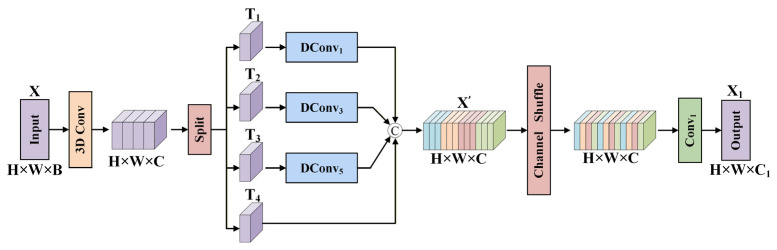
Details of the MsFEF module.

Considering the diverse morphology of bacterial colonies and the complex spectral responses in HSI, we propose a multi-scale feature enhancement and fusion (MsFEF) module, which is shown in Figure 2. This module fully extracts and fuses spatial–spectral information from the input HSI data through a hierarchical feature processing strategy, enhancing the overall representational capability of the features. At the same time, it provides efficient feature representations for the subsequent encoders to capture complex spatial–spectral relationships.

Specifically, for the input HSI data $X\in {\mathbb{R}}^{H\times W\times B}$, we first apply a 3D convolution to perform shallow feature extraction. Then, along the channel dimension, the features are divided into four sub-features $\left\{T_{1},T_{2},T_{3},T_{4}\right\}$, and multi-scale convolution operations are applied to the first three sub-features. We use depth-wise convolution instead of traditional convolution to better reduce the model’s computational cost in terms of parameters. Multi-scale convolutions capture features information at different scales, helping to extract both local and global features in the image, thus enabling multi-scale perception. We use three parallel depth-wise convolution blocks, with kernel sizes of 1×1, 3×3, and 5×5. Each convolutional block consists of a depth-wise convolution layer, a batch normalization (BN) layer, and a Gaussian error linear unit (GELU) activation layer. To integrate features from different scales and enhance the model’s contextual awareness, we concatenate the multi-scale branch output features with the fourth sub-feature $T_{4}$ (which retains the original shallow features) along the channel dimension, resulting in an initial fused feature map $X^{\prime }$. The computational process is formalized as:

(1) $$\left(T_{1},T_{2},T_{3},T_{4}\right)=Split\left(3DConv\left(X\right)\right)$$

(2) $$X^{\prime }=Concat\left(DConv_{k}\left(T_{j}\right),T_{4}\right),j\in \left\{1,2,3\right\}$$

where $Split\left(\cdot \right)$ denotes splitting along the channel dimension, $3DConv\left(\cdot \right)$ represents the 3D convolution, $Concat\left(\cdot \right)$ denotes concatenating the feature maps along the channel dimension. $DConv_{k}\left(\cdot \right)$ denotes a depth-wise convolution block with k×k kernel.

Since the aforementioned multi-scale convolutions ignore cross-channel feature interactions, we introduce a channel reshuffling mechanism. By reorganizing the feature channels, we allow information flow between different groups. Specifically, we divide the concatenated feature map $X^{\prime }\in {\mathbb{R}}^{H\times W\times C}$ along the channel dimension into 4 groups, each containing C/4 channels. After adjusting the channel dimension to 4×C/4, we transpose the group order, so that the channels originally belonging to the same group are evenly distributed into different new groups. Finally, we apply a pointwise convolution to enhance feature expression further and perform channel compression, producing the output feature map. This process is formalized as:

(3) $$X_{1}=Conv_{1}\left(Shuffle\left(X^{\prime },\left[4,C/4,H,W\right]\right)\right)$$

where $Conv_{1}\left(\cdot \right)$ indicates a 1×1 convolution, and $Shuffle\left(\cdot \right)$ refers to the channel reshuffling operation.

The final output feature map $X_{1}$ retains both the spatial–spectral multi-resolution information captured by the multi-scale convolutions and the nonlinear deep fusion of features achieved through cross-channel reorganization and pointwise convolutions. This provides a more discriminative feature representation for subsequent encoders to model complex spatial–spectral correlations.

### 3.3. Spatial–Spectral Dual-Branch Feature Enhancement

#### 3.3.1. S^2^DBA

In hyperspectral image classification, accurately capturing the key features of spatial structure and spectral information, and exploring their complementary relationship, is crucial for improving model performance. However, the mainstream spatial and spectral attention mechanisms show certain limitations in hyperspectral image classification. Spatial attention values describe the correlation between pixels but overlook the spectral variations within each pixel. The magnitude of spectral attention cannot scale with the labeled spatial size, thereby limiting the distribution of information [43,44]. To address this issue, we designed the spatial–spectral dual-branch attention (S^2^DBA) module. This module constructs spatial and spectral attention branches in parallel, performing feature weighting from two dimensions: spatial correlation and spectral dependence. The spatial branch generates position-sensitive weights based on local spatial structures, such as colony morphology, while the spectral branch generates band selection weights based on spectral differences between bacteria. Ultimately, through element-wise fusion of the dual-branch weights, the spatial regions and their corresponding spectra are collaboratively enhanced.

**Figure 3:**
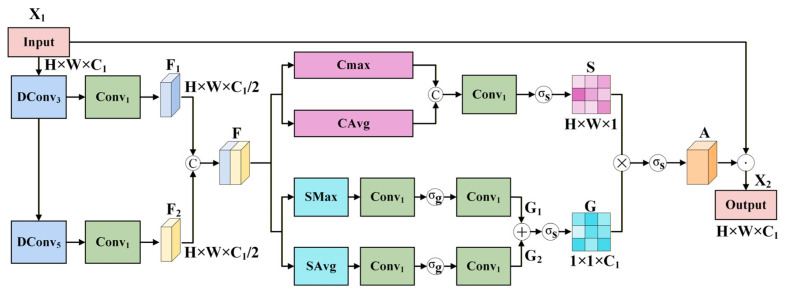
Details of the S^2^DBA module.

The module is shown in Figure 3. Inspired by a novel attention-enhanced network (AENet) [45], which combines large kernel convolutions and attention mechanisms to improve global information extraction, we cascade deep convolutions with increasing dilation rates. Two deep convolutions are used to process the input $X_{1}\in {\mathbb{R}}^{H\times W\times C_{1}}$, and then pointwise convolutions integrate channel information and unify dimensions, yielding low-level features $F_{1}$ with rich spatial detail and high-level features $F_{2}$ with deep channel information. The resulting features $F_{1},F_{2}\in {\mathbb{R}}^{H\times W\times C_{1}/2}$ are concatenated to form the final feature representation F. The process is formalized as follows:

(4) $$F_{1}=Conv_{1}\left(DConv_{3}\left(X_{1}\right)\right)$$

(5) $$F_{2}=Conv_{1}\left(DConv_{5}\left(DConv_{3}\left(X_{1}\right)\right)\right)$$

(6) $$F=Concat\left(F_{1},F_{2}\right)$$

Next, we employ a parallel spatial–spectral selection mechanism to assign attention weights dynamically. Specifically, the extracted feature map F is fed into parallel spatial and spectral attention branches to obtain the corresponding weights. In the spatial attention branch, max pooling and average pooling are applied along the channel dimension to reduce the dimensionality and focus on spatial features. To facilitate information exchange between different spatial feature maps, the two pooled features are concatenated along the channel dimension and then fused using a convolutional layer. A Sigmoid activation function $\sigma_{s}\left(\cdot \right)$ is applied to generate the spatial attention weights $S\in {\mathbb{R}}^{H\times W\times 1}$. This process is formalized as:

(7) $$S=\sigma_{s}\left(Conv_{1}\left(Concat\left(CMax\left(F\right),CAvg\left(F\right)\right)\right)\right)$$

where $CMax\left(\cdot \right)$ and $CAvg\left(\cdot \right)$ represent channel-wise max pooling and average pooling operations, respectively.

In the spectral attention branch, spatial max pooling $SMax\left(\cdot \right)$ and average pooling $SAvg\left(\cdot \right)$ are used to capture both global trends and local peaks in the spectral channels. Each of the two pooled feature maps is processed by a 1×1 convolution that reduces the number of channels from *C* to *C/α*, where *α* is the reduction ratio, followed by a nonlinear transformation and another 1×1 convolution to restore the original channel dimension. This compression-expansion structure allows the model to focus on the most discriminative spectral features. The two transformed features are added and then passed through a Sigmoid activation function to generate the spectral attention weights. This process is formalized as:

(8) $$G_{1}=Conv_{1}\left(\sigma_{g}\left(Conv_{1}\left(SMax\left(F\right)\right)\right)\right)$$

(9) $$G_{2}=Conv_{1}\left(\sigma_{g}\left(Conv_{1}\left(SAvg\left(F\right)\right)\right)\right)$$

(10) $$G=\sigma_{s}\left(G_{1}+G_{2}\right)$$

where $\sigma_{g}\left(\cdot \right)$ denotes the GELU activation function.

The spatial–spectral selection weights are obtained through matrix multiplication (⊗), followed by a Sigmoid activation function to generate the final attention weights:

(11) $$A=\sigma_{s}\left(S⊗G\right)$$

The S^2^DBA module then performs element-wise multiplication (⊙) between the input feature *X*_1_ and the attention weights *A* to produce the final output:

(12) $$X_{2}=X_{1}⊙A$$

#### 3.3.2. Pixel Embedding

For the output $X_{2}$ of the S^2^DBA module, we need to encode it into a sequence of tokens for subsequent processing by the FSDE module. Traditional vision transformers [33] use patch embedding to achieve this. However, while patch embedding improves computational efficiency, it also results in the loss of fine-grained details, which can lead to mixed-pixel interference. Given the uneven distribution of bacteria in wound infection regions, where colonies often appear small and have irregular shapes, we adopt pixel embedding to enable more precise spatial–spectral joint modeling of bacterial colonies. Specifically, we apply a convolutional layer to map $X_{2}\in {\mathbb{R}}^{H\times W\times C_{1}}$ to a new feature space ${\mathbb{R}}^{H\times W\times d}$, and then flatten it into spatial–spectral tokens $X_{3}\in {\mathbb{R}}^{n\times d}$, where n=H×W and d denote the number of tokens and embedding dimension, respectively.

### 3.4. Hierarchical Dual-Domain Encoding Learning and Classification

In the step 3, the spatial–spectral feature tokens produced in the step 2 are first processed by stacked frequency and spatial–spectral domains encoding modules for hierarchical feature learning, then classified through a fully connected layer. Detailed descriptions of each component follow.

#### 3.4.1. FSDE

**Figure 4:**
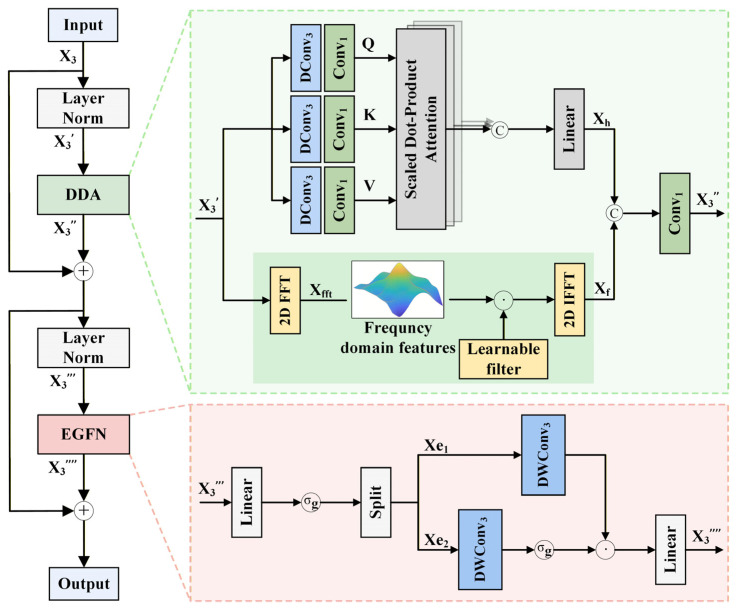
Details of the FSDE module.

The proposed frequency and spatial–spectral domains encoding (FSDE) module is primarily composed of the dual-domain attention (DDA) mechanism and the enhanced gated feedforward network (EGFN), as shown in Figure 4. Below, we provide a detailed explanation of each component.

The DDA is designed as a dual-path structure consisting of two branches. One branch applies a Fourier transform to the feature tokens for frequency domain learning, while the other branch processes feature in the spatial–spectral domain using a CNN-Transformer.

Specifically, in the frequency domain attention branch, we first reshape the given spatial–spectral tokens $X_{3}^{\prime }\in {\mathbb{R}}^{n\times d}$ into ${\mathbb{R}}^{n\times p\times p}$, and apply a Fourier transform $F\left(\cdot \right)$ to obtain the representation of X in the frequency domain:

(13) $$X_{fft}=F\left(X_{3}^{\prime }\right)$$

As demonstrated by Rao et al. [46], the multiplication in the frequency domain is equivalent to convolution in the spatial domain. Therefore, we use a learnable filter *L* [47] to simulate the interaction between tokens. We then perform element-wise multiplication between the frequency domain features $X_{fft}$ and the filter *L* to reconstruct the frequency features. Finally, the inverse Fourier transform $F^{-1}\left(\cdot \right)$ is applied to the reconstructed frequency features to map them back to the spatial domain, obtaining the updated tokens:

(14) $$X_{f}=F^{-1}\left(X_{fft}*L\right)$$

In the spatial–spectral attention branch, we first flatten the given spatial–spectral tokens $X_{3}^{\prime }$ into ${\mathbb{R}}^{H\times W\times d}$. While traditional methods use positional encoding to provide location information within sequences, recent studies [48,49] have shown that convolution operations can implicitly encode positional information. Therefore, we apply depth-wise separable convolution to multi-head attention, which not only enhances the model’s ability to capture contextual information but also significantly reduces the number of parameters compared to fully connected layers. Through convolutional operations, $X_{3}^{\prime }$ is mapped to three vectors: the query vector *Q*, key vector *K*, and value vector *V*, where $Q,K,V\in {\mathbb{R}}^{n\times d}$. These vectors are then split along the channel dimension into *h* independent attention heads, each with a dimensionality of $d_{head}=d/h$ and $Q_{i},K_{i},V_{i}\in {\mathbb{R}}^{n\times d_{head}}$:

(15) $$\left\{\begin{array}{l} Q=\left\{Q_{1},Q_{2},\cdots ,Q_{i},\cdots ,Q_{h}\right\}\\ K=\left\{K_{1},K_{2},\cdots ,K_{i},\ldots ,K_{h}\right\}\\ V=\left\{V_{1},V_{2},\cdots ,V_{i},\cdots ,V_{h}\right\} \end{array}\right.$$

Attention scores are then computed independently for each head:

(16) $$Z_{i}=Attention\left(Q_{i},K_{i},V_{i}\right)=Softmax\left(\frac{Q_{i}K_{i}^{T}}{\sqrt{d_{head}}}\right)V_{i}$$

Next, the attention outputs $Z_{i}\in {\mathbb{R}}^{n\times d_{head}}$ from all heads are concatenated to restore the original high-dimensional feature representation, followed by a linear projection layer to produce the final multi-head attention output:

(17) $$X_{h}=Concat\left(Z_{1},Z_{2},\cdots ,Z_{i},\cdots Z_{h}\right)W$$

where W represents the learnable weights.

Finally, the frequency domain features and spatial–spectral domain features are fused to obtain the dual-domain feature output:

(18) $$X_{3}^{\prime \prime }=Conv_{1}\left(Concat\left(X_{f},X_{h}\right)\right)$$

To address the redundancy in feature channels, we design EGFN to modulate features adaptively. The input $X_{3}^{\prime \prime \prime }\in {\mathbb{R}}^{n\times d}$ is first passed through a nonlinear mapping and then evenly split along the channel dimension into two parts $X_{e1},X_{e2}\in R^{n\times d/2}$. A depth-wise separable convolution is applied to the $X_{e1}$ branch, while the $X_{e2}$ branch undergoes a depth-wise separable convolution followed by a GELU activation to generate a dynamic modulation signal. The two processed branches are then multiplied element-wise to produce a richer feature representation, which is finally projected back to the original dimension via a linear layer. This process can be formalized as:

(19) $$\left(X_{e1},X_{e2}\right)=Split\left(\sigma_{g}\left(Linear\left(X_{3}^{\prime \prime \prime }\right)\right)\right)$$

(20) $$X_{3}^{⁗}=Linear\left(DWConv_{3}\left(X_{e1}\right)⊙\sigma_{g}\left(DWConv_{3}\left(X_{e2}\right)\right)\right)$$

where $DWConv_{3}\left(\cdot \right)$ denotes depth-wise separable convolution.

#### 3.4.2. Classifier

After the final FSDE module, a global average pooling operation is applied to aggregate spatial dimensions. A fully connected layer is then used to generate the final classification output $Y\in {\mathbb{R}}^{1\times 8}$.

## 4. Experiment and Discussion

### 4.1. Sample Preparation

#### 4.1.1. Bacterial Preparation

The experiment targeted eight common pathogenic bacteria associated with clinical wound infections, including *Acinetobacter baumannii* (AB), *Staphylococcus epidermidis* (SE), *Proteus vulgaris* (PV), *Escherichia coli* (EC), *Staphylococcus saprophyticus* subsp. *saprophyticus* (SS), *Klebsiella pneumoniae* (KP), *Staphylococcus aureus* (SA), and *Pseudomonas aeruginosa* (PA). All bacterial samples were provided by Chongqing Jinmai Biotechnology Co., Ltd. (Chongqing, China). Eight types of bacteria were separately prepared in four concentrations (10^4^ CFU/mL, 10^5^ CFU/mL, 10^7^ CFU/mL, and 10^9^ CFU/mL).

#### 4.1.2. Animal Preparation

Forty-eight male SD mice aged 4 to 6 weeks, with an average weight of 180–220 g, were selected and provided by the Animal Center (Laboratory Animal Production License No. SCXK (Yu) 2022-0011). All related animal experiments were conducted at the Army Specialty Medical Center. Experimental procedures strictly adhered to the Regulations on the Administration of Laboratory Animals and institutional ethical standards. The experimental protocol was approved by the Laboratory Animal Welfare and Ethics Committee of the Army Medical University (Laboratory Animal Use License No. SYXK (Yu) 2022-0018). After hair removal and cleansing, two full-thickness skin wounds (diameter: 1.5 cm, spacing: ~2 cm) were created on both sides of the spine of each mouse using a custom-made punch.

#### 4.1.3. Bacterial Inoculation

**Figure 5:**
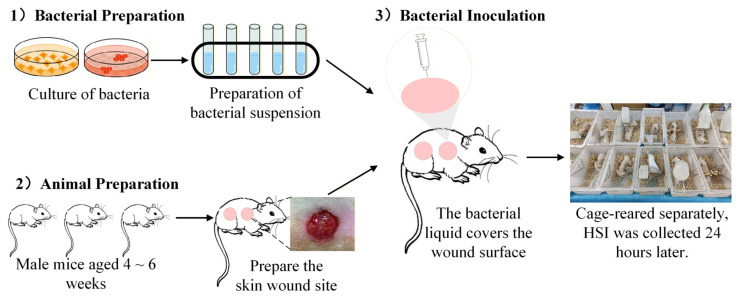
Overall experimental workflow of sample preparation.

Hemostasis was achieved by compression after wound creation. Each bacterial strain was inoculated into six mice. For each mouse, four wounds were inoculated with four different concentrations of the same type of bacterial solution. For each wound, 200 μL of bacterial suspension was applied and evenly spread using a sterile applicator. After wound treatment, mice were housed individually in cages under controlled conditions with an ambient temperature of 20–24 °C, with free access to food and water for 24 h. The sample preparation procedure is illustrated in Figure 5.

### 4.2. Hyperspectral Image Acquisition and Processing

**Figure 6:**
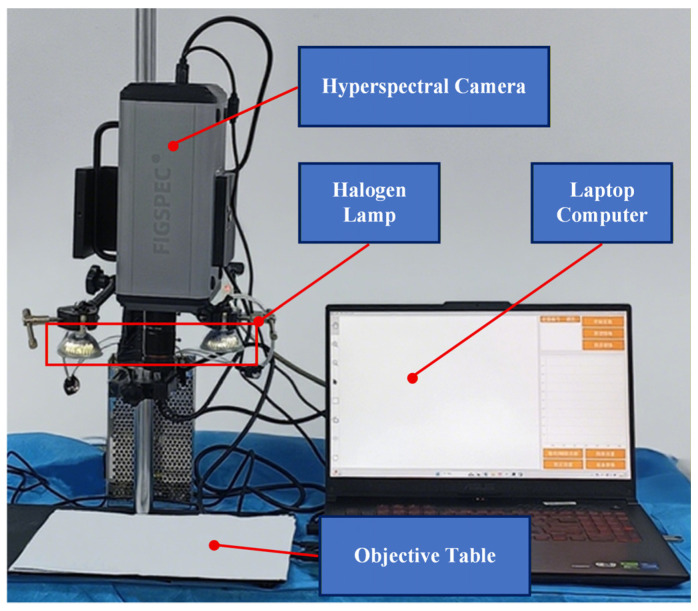
Hyperspectral image acquisition system.

In this experiment, hyperspectral images of individual wound sites were collected using a visible-near-infrared (VIS-NIR) hyperspectral imaging system, resulting in a total of 192 images. The hyperspectral imaging system consisted of a FigSpec Hyperspectral Camera FS-22 (FigSpec Technology (Zhejiang) Co., Ltd, Hangzhou, China), a Windows 11 operating system, and two 50W halogen light sources. The setup of the hyperspectral image acquisition system is illustrated in Figure 6.

During image acquisition, mice were placed on the specimen stage, with each wound positioned directly beneath the camera lens to ensure complete coverage of the wound area. The hyperspectral images cover a spectral range of 400–1000 nm with a spectral resolution (FWHM) of 5 nm and a sampling interval of 1 nm, resulting in 600 original spectral channels. Due to the high correlation and redundancy among adjacent spectral bands in hyperspectral data, the first step is to merge channels to integrate some spectral bands, in order to reduce spectral redundancy while retaining discriminative information, thereby improving the stability of feature representation and reducing the computational complexity in subsequent steps. After channel merging, a hyperspectral image with 300 spectral bands is obtained. To assess the rationality of the selected spectral dimensions, we conducted a sensitivity analysis under different channel merging coefficients. The relevant details can be found in Section 4.6.4.

During the acquisition of hyperspectral data, changes in illumination and background interference can easily introduce uncertainties. To reduce the impact of sensor dark noise and illumination fluctuations on data quality, this paper performs reflection normalization on the original data based on black-and-white calibration. By introducing white reference and dark reference images, the original image is converted into a reflectance form, and its expression is as follows:

(21) $$H_{r}=\frac{H_{o}-H_{b}}{H_{w}-H_{b}}$$

where $H_{o}$ is the originally collected hyperspectral image, $H_{w}$ representing the white reference image obtained through a standard white board, $H_{b}$ is the dark reference image obtained under the condition of a blocked lens.

### 4.3. Wound Bacteria HSI Dataset

**Figure 7:**
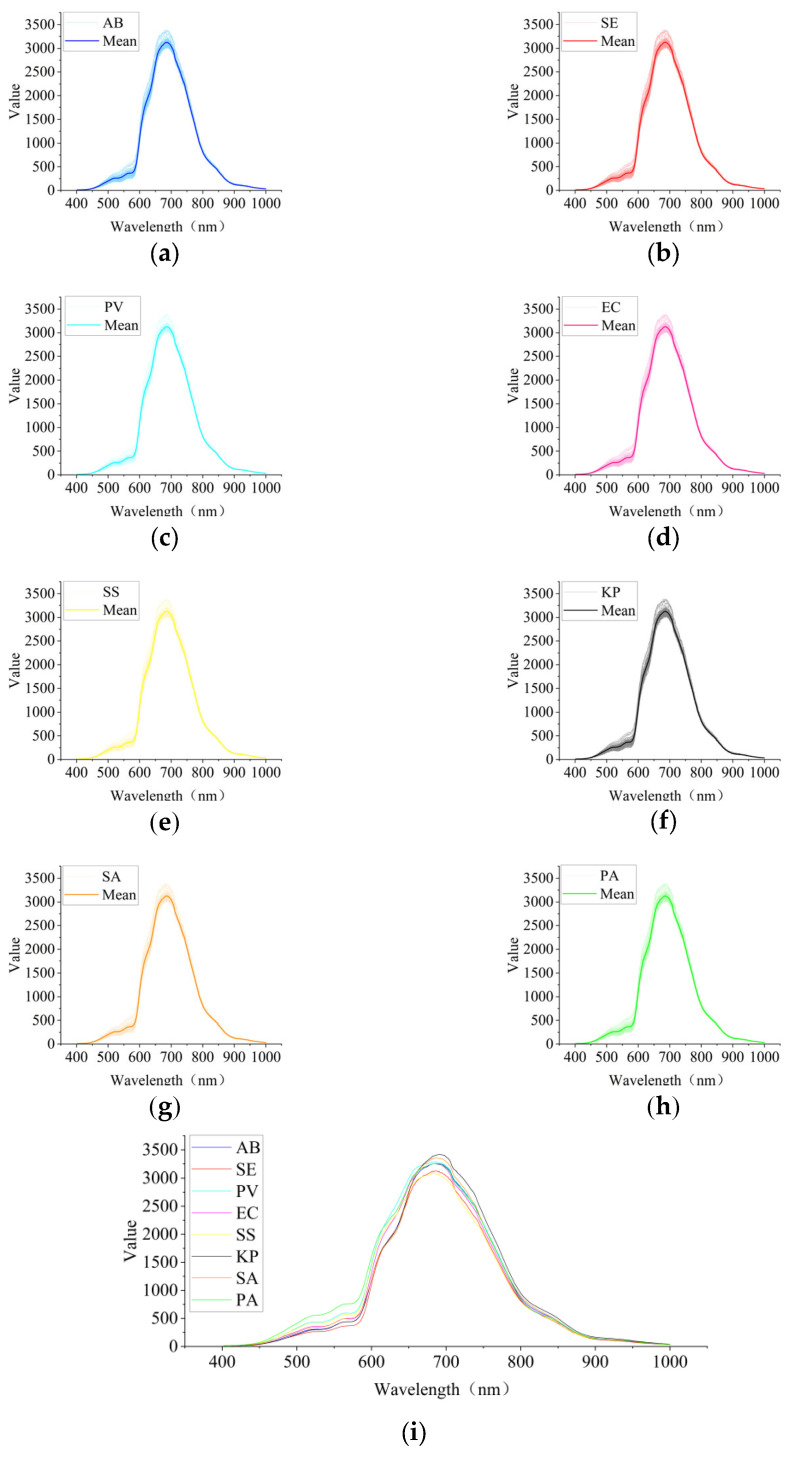
Comparison of pixel-wise reflectance spectra and mean spectra within ROIs from wounds infected by different bacteria. (**a**–**h**) Pixel-level reflectance spectra within a single 10 × 10 ROI and the corresponding mean reflectance spectrum for wounds infected by eight different bacterial species (AB, SE, PV, EC, SS, KP, SA, and PA), where the thin curves represent the reflectance spectra of individual pixels within the ROI and the thick curve denotes the mean reflectance spectrum of the ROI. (**i**) Comparison of the mean reflectance spectra of wounds infected by different bacterial species after Savitzky–Golay smoothing. Subsequently, to suppress random noise in the hyperspectral data, the Savitzky–Golay (SG) algorithm (with window_length set to 7 and polyorder set to 2) was applied to smooth the pixel-level spectra within each image patch. After the above preprocessing, the obtained hyperspectral data *X* is used as the input for the subsequent network model. Based on these ROIs, the mean reflectance spectra of wounds infected by eight different bacterial species were obtained, as shown in Figure 7i. Although the mean reflectance spectra of different bacteria exhibit similar overall trends across the visible to near-infrared wavelength range, noticeable differences in spectral intensity can still be observed around the main reflectance peak and its adjacent bands.

To extract representative bacterial spectral information from hyperspectral images, the acquired wound hyperspectral images were further processed. First, ENVI 5.6 software was used to manually delineate regions of interest (ROIs) in wound areas where bacterial colonies were relatively dense and uniformly distributed, thereby reducing the interference from background tissue and non-infected regions on spectral analysis. Subsequently, to further construct sample data for model training, a sliding-window approach was applied to the ROIs to generate multiple image patches with a size of 10 × 10 pixels. As shown in Figure 7a–h, the pixel-wise reflectance spectra within a single 10 × 10 ROI and the corresponding mean reflectance spectrum are presented for eight different bacterial infections, where the thin curves represent the reflectance spectra of individual pixels within the ROI and the thick curve denotes the mean reflectance spectrum of the ROI. It can be observed that the spectral curves of different pixels within the same ROI exhibit a high degree of consistency in overall shape and variation trend, indicating that the spectral characteristics within this region are relatively stable. Meanwhile, certain levels of dispersion can still be observed in specific local bands, reflecting the influence of non-uniform bacterial distribution and microstructural heterogeneity of the wound tissue on spectral responses.

**Figure 8:**
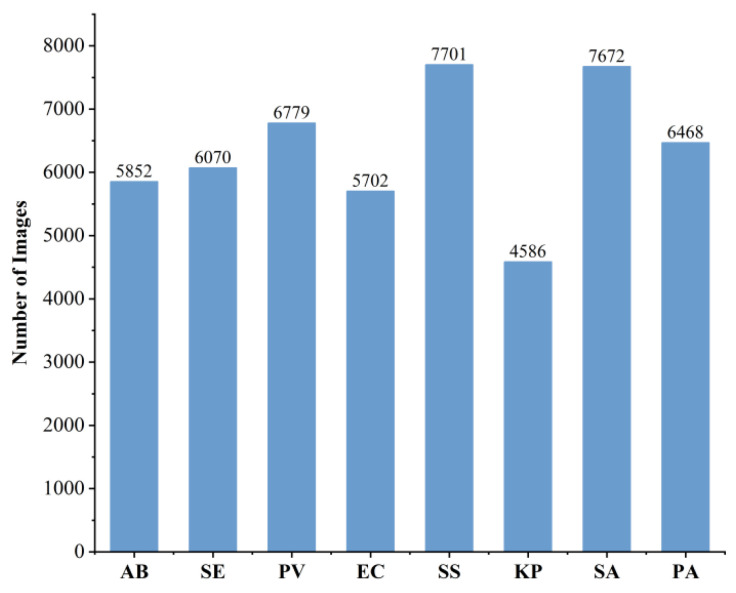
Sample size statistics for each bacterial species.

The classification results for the treated data, based on bacterial strains, are shown in Figure 8. A total of eight bacterial categories were identified. The following image counting results were thus obtained: AB (5852 images), SE (6070 images), PV (6779 images), EC (5702 images), SS (7701 images), KP (4586 images), SA (7672 images), and PA (6468 images), totaling 50,830 images. It can be seen that the number of samples in the KP category is significantly less than that of other categories. This imbalance in data distribution during the model training process may introduce bias, causing the model to be more inclined to learn the features of the categories with a larger number of samples, thereby limiting its discriminative ability and generalization performance for the categories with a smaller number of samples. To address this issue, we adopted data augmentation methods such as random rotation for the KP category, increasing the spatial diversity of the samples without compromising the spectral information. Through the above augmentation operations, the number of samples in the KP category increased from 4586 to 5886, making it closer in scale to the other categories.

In order to evaluate the stability and robustness of the proposed method in the task of hyperspectral recognition of wound bacteria, this paper adopted a multi-round cross-validation strategy based on individual animals in the experimental evaluation stage. Specifically, for each bacterial category, the experimental data came from 6 different mouse individuals. We used the mouse individuals as the smallest partition unit. In each round of the experiment, the data of 4 mice were used as the training set, 1 mouse’s data as the validation set, and the remaining 1 mouse’s data as the test set, forming a 4:1:1 partition of the training set, validation set, and test set. By rotating different mouse individuals as the test objects, a total of 6 independent experiments were conducted. It should be emphasized that all the hyperspectral images and the patches extracted through the sliding window corresponding to the same mouse were strictly assigned to the same data subset.

### 4.4. Experimental Setup and Evaluation Metrics

The proposed MDF2Former was implemented using the PyTorch 2.2.0 framework and deployed on an NVIDIA GeForce RTX 4070 GPU with 8GB of memory. The training process utilized the Adam optimizer with an initial learning rate set to 0.001. The loss function used was cross-entropy loss, with a batch size of 32, and the model was trained for a total of 50 epochs.

To evaluate the classification performance of the model, we used Accuracy, Precision, Recall, F1-score, and Kappa coefficient as key metrics for assessing bacterial identification results. These metrics provide a comprehensive evaluation of model performance by measuring the agreement between the predicted results and the actual labels. Each evaluation metric is presented as a percentage, ranging from 0% to 100%. Among them, Accuracy measures the model’s overall ability to correctly classify all samples, reflecting its comprehensive performance across all classes. Precision indicates the proportion of true positive predictions among all samples predicted as positive, highlighting the model’s ability to avoid false positives. Recall reflects the model’s ability to correctly identify actual positive samples, with a focus on its capacity to avoid false negatives. F1-score is the harmonic mean of Precision and Recall, used to balance both aspects. Kappa coefficient evaluates the agreement between the model’s predictions and the true labels. A higher Kappa value indicates more reliable model performance. These metrics are calculated using the following formulas:

(22) $$Accuracy=\frac{TP+TN}{TP+TN+FP+FN}$$

(23) $$Precision=\frac{TP}{TP+FP}$$

(24) $$Recall=\frac{TP}{TP+FN}$$

(25) $$F1‐Score=2\times \frac{Precision\times Recall}{Precision+Recall}$$

(26) $$Ka=\frac{\left(TP+FP\right)\times \left(TP+FN\right)+\left(FN+TN\right)\times \left(FP+TN\right)}{{\left(TP+FN+FP+TN\right)}^{2}}$$

(27) $$Kappa=\frac{Accuracy-Ka}{1-Ka}$$

where *TP* represents the number of actual positive and correctly predicted as positive, *TN* represents the number of actual negative and correctly predicted as negative, *FP* represents the number of actual negative but incorrectly predicted as positive, and *FN* represents the number of actual positive but incorrectly predicted as negative.

It should be noted that all samples in this study fall into predefined bacterial categories. In this setting, true negatives (TN) do not correspond to clinically non-infected cases and are used solely for evaluation metric computation.

### 4.5. Experimental Results Analysis

#### 4.5.1. Comparison Algorithm Setup

To verify the effectiveness of the proposed network for hyperspectral image classification and detection of wound bacteria, we selected eight representative hyperspectral image classification methods for comparison. These include two CNN-based methods: going deeper with contextual CNN (CDCNN) [50], a 2D convolutional neural network, and spectral-spatial residual network (SSRN) [51], a 3D convolutional neural network; three Transformer-based methods: ViT [33], spectral transformer (SpectralFormer) [35], and SS1DSwin [41]; and three hybrid CNN-Transformer approaches: spectral-spatial feature tokenization transformer (SSFTT) [52], bottleneck spatial–spectral transformer (BS2T) [53], and grouped multi-attention network (GMANet) [54]. All methods were evaluated on the wound bacteria HSI dataset constructed in this study.

#### 4.5.2. Experimental Results

The experimental results are summarized in Table 1, with the best values highlighted in bold and the second-best values underlined. As shown in the table, our proposed MDF2Former achieved the highest recognition accuracy on the wound bacteria HSI dataset. Specifically, the model attained an Accuracy of 91.94%, Precision of 92.26%, Recall of 91.94%, F1-score of 92.01%, and a Kappa coefficient of 90.73%. These results highlight the superior performance of MDF2Former over other state-of-the-art hyperspectral image classification algorithms. Furthermore, by analyzing the standard deviation, it can be observed that the proposed method maintains a relatively low performance fluctuation among different mouse individuals, indicating that the model has good stability and robustness.

In general, methods based on 2D CNNs performed relatively poorly on the wound bacteria HSI dataset. This result may be attributed to the nature of bacterial classification tasks, which heavily rely on spectral fingerprint information. While 3D CNNs provide some improvements, their overall performance remains limited due to CNNs’ insufficient ability to extract fine-grained spectral features and capture inter-spectral correlations. In contrast, Transformer-based methods generally outperformed CNN-based approaches on this dataset, indicating that the self-attention mechanism and sequence modeling capabilities of Transformers are effective for processing high-dimensional spatial–spectral representations. Hybrid CNN-Transformer methods demonstrated better performance, benefiting from a hierarchical structure that combines local feature extraction with global context modeling.

#### 4.5.3. Error Analysis

**Figure 9:**
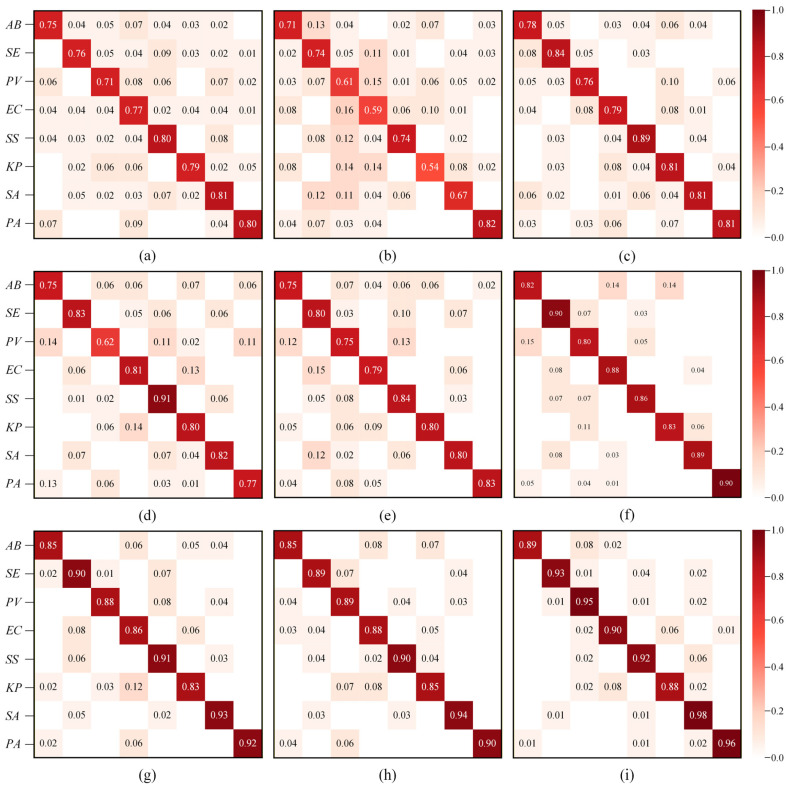
The confusion matrix results are (**a**) SSRN, (**b**) CDCNN, (**c**) ViT, (**d**) Spectral Former, (**e**) SS1DSwin, (**f**) SSFTT, (**g**) BS2T, (**h**) GMANet, and (**i**) MDF2Former.

To more intuitively present the misclassification patterns of different bacterial species in the wound bacteria HSI classification task, we visualized the model outputs using confusion matrices. The confusion matrix results for all models are shown in Figure 9. From the perspective of per-class classification accuracy, our proposed model demonstrates a clear advantage, as it achieves the highest single-class accuracy rate among all 8 types of bacteria. For Klebsiella pneumoniae, its performance is second only to GMANet. As observed from the confusion matrices in Figure 9a–h, other models commonly suffer from a high proportion of misclassified samples and scattered misclassification across multiple classes. This multi-class cross-interference with a single class is reflected by the high distribution of values outside the diagonal of the confusion matrix, indicating insufficient bacterial recognition ability. In contrast, as shown in Figure 9i, our model results in fewer misclassification instances, with most errors occurring between bacteria of the same genus or family. For instance, approximately 8% of the KP samples were misclassified as EC, and around 6% of the EC samples were misclassified as KP. This phenomenon can be attributed to the fact that both belong to the Enterobacteriaceae family and share similar metabolic product characteristics and partially overlapping spectral response patterns, which increases the difficulty of discrimination. Similarly, a certain degree of confusion was also observed within the Staphylococcus genus, such as 4% of the SE samples being misclassified as SS, 2% as SA, and there were also a few misclassifications between SS and SA.

**Figure 10:**
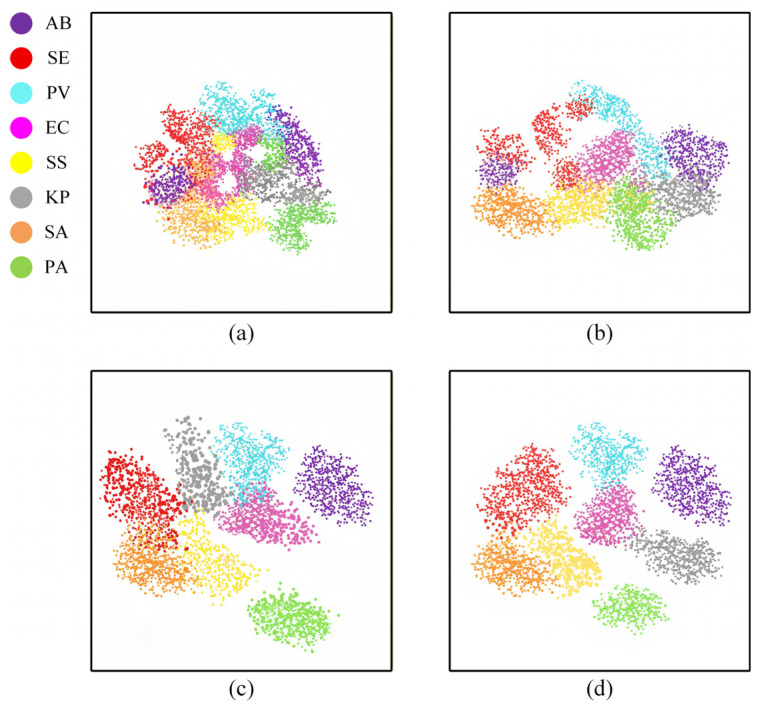
The t-SNE visualization of the learned features from (**a**) SSRN, (**b**) ViT, (**c**) BS2T and (**d**) MDF2Former.

To further provide quantitative support for the above misclassification phenomenon from the perspective of feature representation, we introduce t-SNE visualization to conduct dimensionality reduction analysis on the high-dimensional features before the classification decisions of different models. As shown in Figure 10, we select four representative methods: SSRN, ViT, BS2T, and the proposed MDF2Former. From the visualization results, it can be seen that SSRN exhibits a significant category overlap phenomenon in the feature space, with the feature distributions of different categories highly intersecting. ViT has improved in the overall organization of features, but there is still a significant overlap between multiple categories. In contrast, BS2T has improved in both intra-class clustering and inter-class separation, but there is still some feature mixing for certain bacterial categories. The proposed MDF2Former forms a more compact intra-class clustering structure in the feature space and establishes clearer inter-class discrimination boundaries. It should be noted that bacteria belonging to the same genus or family still maintain a reasonable proximity relationship in the feature space, but the degree of overlap has significantly decreased. This distribution characteristic is highly consistent with the misclassification patterns observed in the confusion matrix.

### 4.6. Ablation Study

It should be noted that the ablation experiments are designed to analyze the relative contributions of each module of the model. Therefore, they are conducted under the same data partition settings to ensure the fairness of the comparison.

#### 4.6.1. Module Disassembly Ablation Study

To comprehensively analyze the proposed model, we designed three ablation comparisons to study the effectiveness of the MsFEF module, S^2^DBA module, and FSDE module, respectively. The experimental results are shown in Table 2. All ablation experiments were conducted on the wound bacteria HSI dataset, and three evaluation metrics were used to assess their performance. For Model 1, we removed the feature extraction module and used only one 3D convolution and one 2D convolution for feature extraction. For Model 2, we replaced the encoder module with the Transformer structure from ViT. For Model 3, we removed the attention module, and each model differs by only one component.

As shown in the table, compared to Model 1 achieved improvements of 15.67% in Accuracy, 14.45% in Precision, and 15.67% in Recall. Compared to Model 2, Model 3 improved by 8.71% in Accuracy, 9.68% in Precision, and 8.71% in Recall. This indicates that combining global information encoding via transformer on top of multi-scale convolution feature extraction results in complementary synergy between the two modules, which is more beneficial for improving classification performance. Additionally, comparing Model 1 and Model 2 shows that the S^2^DBA module realizes its full potential only when combined with MsFEF. S^2^DBA allocates attention weights to the multi-scale features output by MsFEF through a spatial–spectral dual-branch attention mechanism, thereby enhancing the expression of key features. Therefore, when an S^2^DBA module is added on top of Model 3, the proposed model achieves the best classification performance, with Accuracy, Precision, and Recall reaching 92.22%, 92.60%, and 92.22%, respectively.

Thus, the ablation study results validate the rationality and effectiveness of each module in the proposed model, clearly demonstrating that each component contributes to classification performance to varying degrees and that the overall model exhibits strong advantages.

#### 4.6.2. Influence of FSDE Module Quantity

The configuration of the number of FSDE modules across the three different scales of feature maps is a key parameter for controlling multi-scale feature encoding capabilities. Since the number of modules affects the learning intensity at each hierarchical level and there is no prior knowledge about the contribution of each scale to the classification task, we optimized this setting experimentally. To balance model complexity and representational power, we limited the number of FSDE modules per scale to a maximum of 2, resulting in eight different combinations for evaluation.

As shown in Table 3, the best-performing configuration is highlighted in bold. When the module quantity is set to [1,2,1], MDF2Former achieved optimal performance, with an Accuracy of 92.06%, Precision of 92.22%, and Recall of 92.60%. In contrast, the suboptimal settings of [1,1,1] and [2,1,1] yielded Accuracy values of 87.52% and 88.34%, respectively. These results suggest that increasing the number of modules at the middle scale contributes more to performance gains than increasing those at the shallow or deep scales. Assigning two FSDE modules to the middle-scale feature map enables deeper extraction of mid-level semantic features, while assigning 1 module each to the other two scales preserves fine-grained detail and global context, effectively avoiding feature redundancy and computational overload. Therefore, we selected the [1,2,1] configuration as the optimal setup for MDF2Former.

#### 4.6.3. Impact of FSDE Module Hierarchical Structure

To further verify the role of the hierarchical structure we designed in the FSDE module, we compared three different architectural forms: a non-hierarchical structure (where the four FSDE modules are directly connected in series), a two-level hierarchical structure (with the module configuration as [2, 2]), and a three-level hierarchical structure (i.e., the configuration proposed in this paper [1,2,1]). It should be emphasized that to ensure the fairness of the experimental comparison, the total number of FSDE modules in the three architectures is fixed at 4, with only the organization of the modules and the hierarchical division differing.

The experimental results are shown in Table 4. It can be observed that the model with the hierarchical structure outperforms the non-hierarchical model in all indicators. Among them, the proposed three-level hierarchical structure has improved the accuracy, precision, and recall rates by 10.70%, 9.97%, and 10.70%, respectively, compared to the non-hierarchical structure. This significant improvement indicates that the hierarchical structure of the FSDE module plays a crucial role in enhancing the feature expression ability of the model. Specifically, the hierarchical structure can model the feature interaction relationships at different scales in a step-by-step manner, enabling the model to simultaneously consider local details and global context information. Compared to the non-hierarchical structure that stacks all FSDE modules on a single scale, the hierarchical structure effectively enhances the model’s ability to model multi-scale spatial–spectral features through the stepwise downsampling and encoding of multi-scale feature maps. Moreover, the introduction of multi-scale feature maps reduces the computational complexity of the model to a certain extent. Therefore, this experimental result proves the advantages of the proposed hierarchical structure in balancing the model’s representation ability and computational efficiency.

#### 4.6.4. Sensitivity Analysis of Spectral Dimensionality

To further validate the rationality of the selected merging factor, a sensitivity analysis was conducted under different spectral merging configurations. The hyperspectral camera operates within the spectral range of 400–1000 nm and provides 600 original bands, corresponding to an initial sampling interval of approximately 1 nm and a spectral resolution (FWHM) of 5 nm. From the perspective of signal sampling, the sampling interval should be substantially smaller than the spectral resolution; meanwhile, from a spectral coverage perspective, when the sampling interval approaches the FWHM, only basic overlap between adjacent spectral responses can be guaranteed. Based on these considerations, merging factors of 1, 2, 3, 4, 5, and 6 were selected for comparison. All other experimental settings remained unchanged except for the spectral dimensionality.

The results are summarized in Table 5. Using all 600 bands (merging factor = 1) yields the highest accuracy; however, compared with the configuration using a merging factor of 2, the performance gain is only 0.06%, while the computational cost nearly doubles. As the merging factor further increases, the performance degradation becomes more pronounced. This trend suggests that when the sampling interval approaches or exceeds the spectral resolution, the model’s ability to capture subtle spectral variations is significantly weakened.

Overall, a merging factor of 2 achieves a favorable trade-off between classification performance and computational complexity while maintaining near-sufficient spectral sampling, and is therefore adopted as the default configuration in this study.

### 4.7. Model Complexity Analysis

To evaluate the computational complexity and inference efficiency of our proposed model, we compared the number of trainable parameters, floating-point operations (FLOPs), and inference time with those of other benchmark models. As shown in Table 6, the proposed model has only 0.470M trainable parameters, slightly more than the ViT model. Its FLOPs are merely 47.762M, which is significantly lower than those of the other methods. In terms of inference efficiency, our model exhibits the shortest inference time among all compared models. Therefore, the proposed model achieves superior inference efficiency while maintaining a low number of trainable parameters and minimal computational overhead. These results clearly demonstrate that the proposed model is of low complexity and delivers the best performance in multi-class wound bacteria recognition tasks.

## 5. Conclusions

This paper proposes a multi-scale dual-domain feature fusion transformer network (MDF2Former) for the task of real wound bacterial identification, which is used for high-resolution wound bacterial recognition. This method achieves multi-scale modeling of spatial–spectral features and collaborative representation of local and global dependencies through a hybrid CNN-Transformer framework, effectively overcoming the bottlenecks of traditional models in capturing fine spatial texture features and long-distance spectral modeling.

Extensive experiments on our self-constructed wound bacteria HSI dataset demonstrated the superior performance of MDF2Former compared to other HSI classification algorithms. The model achieved 91.94% Accuracy, 92.26% Precision, 91.94% Recall, 92.01% F1-score, and 90.73% Kappa coefficient, while also exhibiting clear advantages in computational cost and inference speed. These results confirm the strong potential for applying deep learning and hyperspectral imaging technologies to recognize wound bacteria. It should be noted that this method is specifically designed for the bacterial strain identification module, and its applicable premise is that the infection status has been confirmed by other means. In future work, we will further study the multi-bacterial infection situation in real wound environments and consider validating the proposed method in different animal models to systematically evaluate its generalization ability and robustness. At the same time, we will explore a multi-stage framework that combines infection detection with bacterial strain identification, and further optimize the feature decoupling and classification strategies to continuously enhance the clinical applicability of this method.

## Figures and Tables

**Table 1:** Comparative Results of Different Methods for Bacterial Classification on the Wound Bacteria HSI Dataset. All results are averaged over 6-fold animal-level cross-validation. Best results are in bold, second-best are underlined.

Class	CNN-Based Method		Transformer-Based Method			Hybrid CNN-Transformer Method			Ours
	SSRN	CDCNN	ViT	Spectral Former	SS1D Swin	SSFTT	BS2T	GMANet	MDF2 Former
*AB*	74.25 ± 0.94	70.52 ± 0.13	77.52 ± 0.85	75.12 ± 0.91	75.32 ± 0.32	81.94 ± 1.49	84.51 ± 0.12	84. 91 ± 0.06	**89.** **49 ± 0.03**
*SE*	76.15 ± 1.01	74.36 ± 0.92	83.53 ± 0.92	83.13 ± 0.52	79.95 ± 0.52	90.49 ± 0.25	90.01 ± 0.18	89.42 ± 0.29	**9** **2.70 ± 0.40**
*PV*	70.93 ± 0.62	61.25 ± 2.52	76.46 ± 1.35	61.67 ± 0.95	75.45 ± 0.45	80.49 ± 1.92	87.73 ± 0.49	89. 36 ± 0.62	**94.** **6** **2** **± 0.56**
*EC*	77.26 ± 0.24	58.53 ± 0.52	78.91 ± 0.65	80.84 ± 0.56	79.15 ± 0.26	87.79 ± 0.09	86.16 ± 0.24	87.64 ± 0.12	**89.** **79 ± 0.02**
*SS*	80.35 ± 0.58	74.26 ± 1.56	88.84 ± 2.56	90.58 ± 2.49	83.89 ± 2.08	86.49 ± 0.65	90.95 ± 1.09	89.65 ± 0.96	**92.14** **± 0.11**
*KP*	78.65 ± 0.94	54.12 ± 2.38	80.65 ± 0.94	80.07 ± 0.59	79.95 ± 0.94	82.56 ± 0.86	83.49 ± 0.76	8 5.11 ± 0.56	**8** **8.13 ± 0.79**
*SA*	80.59 ± 1.54	67.24 ± 3.77	81.45 ± 2.45	81.67 ± 1.84	80.18 ± 0.94	89.35 ± 0.35	93.49 ± 0.02	9 3.65 ± 0.25	**9** **7.96 ± 0.15**
*PA*	80.26 ± 0.45	82.29 ± 2.56	80.85 ± 1.09	77.46 ± 0.56	83.43 ± 0.26	90.40 ± 0.14	9 2.12 ± 0.15	90.49 ± 0.21	**9** **6.4** **7** **± 0.12**
Accuracy (%)	76.35 ± 0.80	67.43 ± 1.82	82.29 ± 1.56	78.82 ± 1.28	78.86 ± 0.74	84.84 ± 0.75	87.24 ± 0.40	87.49 ± 0.40	**9** **1** **.** **94 ± 0.28**
Precision (%)	76.16 ± 0.82	67.63 ± 1.79	82.85 ± 1.43	79.46 ± 1.25	78.44 ± 0.76	84.68 ± 0.78	87.59 ± 0.42	87.67 ± 0.38	**92.** **26 ± 0.34**
Recall (%)	76.35 ± 0.80	67.43 ± 1.82	82.29 ± 1.56	78.82 ± 1.28	78.86 ± 0.74	84.84 ± 0.75	87.24 ± 0.40	87.49 ± 0.40	**9** **1** **.** **94 ± 0.28**
F1-score (%)	76.12 ± 0.80	67.58 ± 1.79	82.96 ± 1.62	78.65 ± 1.05	78.98 ± 0.73	84.49 ± 0.72	87.29 ± 0.42	87.28 ± 0.41	**92.0** **1 ± 0.36**
Kappa (%)	75.96 ± 0.79	66.36 ± 1.80	80.42 ± 1.49	74.95 ± 1.36	78.29 ± 0.76	83.12 ± 0.74	86.49 ± 0.41	8 6.94 ± 0.40	**90.** **73 ± 0.29**

**Table 2:** Ablation Study Results on the Wound Bacteria HSI Dataset. Best results are in bold.

Model	MsFEF	S^2^DBA	FSDE	Accuracy (%)	Precision (%)	Recall (%)
Model1		√	√	74.52	77.42	74.52
Model2	√	√		81.48	82.19	81.48
Model3	√		√	90.19	91.87	90.19
Ours	√	√	√	**92.** **22**	**92.** **6** **0**	**92.** **22**

**Table 3:** Results of the Proposed Method with Different Numbers of FSSE Modules on the Wound Bacteria HSI Dataset. Best results are in bold.

Numbers	Accuracy (%)	Precision (%)	Recall (%)
[1,1,1]	87.52	87.25	87.52
[2,1,1]	88.34	88.54	88.34
**[1,2,1]**	**92.22**	**92.60**	**92.22**
[1,1,2]	77.54	80.95	77.54
[2,2,1]	79.52	81.56	79.52
[2,1,2]	77.85	79.60	77.85
[1,2,2]	82.52	84.16	82.52
[2,2,2]	87.32	88.01	87.32

**Table 4:** Results of the Proposed Method Using Different Hierarchical Structures on the Wound Bacteria HSI Dataset. Best results are in bold.

Stage	Accuracy (%)	Precision (%)	Recall (%)
Non-hierarchical	81.52	82.63	81.52
Two-stage	87.68	87.45	87.68
Ours	**92.** **22**	**92.** **6** **0**	**92.** **22**

**Table 5:** Performance Under Different Spectral Merging Factors. The configurations in this paper are in bold.

Merge Factor	Bands	Sampling Interval (nm)	Accuracy (%)	FLOPs (M)	Inference Time (ms)
1	600	1	92.28	83.731	10.750
**2**	**300**	**2**	**92.22**	**47.762**	**6.465**
3	200	3	90.06	32.483	4.571
4	150	4	89.48	28.893	4.060
5	120	5	83.53	24.176	3.610
6	100	6	75.67	20.343	2.932

**Table 6:** Complexity and Running Time of Different Methods on the Wound Bacteria HSI Dataset. Best results are in bold.

Method	Parameters (M)	FLOPs (M)	Inference Time (ms)
SSRN	**0.364**	525.432	17.123
CDCNN	1.064	228.815	7.202
ViT	0.411	426.964	13.519
SpectralFormer	0.433	440.964	13.024
SS1DSwin	0.507	427.324	23.117
SSFTT	1.401	96.020	12.267
BS2T	1.477	220.853	13.827
GMANet	1.935	166.995	24.634
Ours	0.470	**47.762**	**6.465**

## Data Availability

The original contributions presented in this study are included in the article. Further inquiries can be directed to the corresponding author.
